# Characterizing the initial diffusion pattern of pandemic (H1N1) 2009 using surveillance data

**DOI:** 10.1371/currents.RRN1151

**Published:** 2010-03-10

**Authors:** Shui Shan Lee, Ngai Sze Wong

**Affiliations:** The Chinese University of Hong Kong

## Abstract

Using notification data, diffusion of pandemic human influenza A (H1N1) 2009 in Hong Kong was explored with geographic information system (GIS) methodology. Point data were displayed and then analysed with interpolation and the application of SaTScan™. Beginning from 6 initial foci, the spatial distribution has remained heterogeneous at the end of the first three months, with students functioning as the main disseminators. Our study showed that routinely collected surveillance data could be effectively used for describing the epidemic, which could support the development of interventions at local levels.

## Introduction

First identified in Mexico and originating from swine host, the novel human influenza (H1N1) 2009, hereafter referred as swine flu, has rapidly swept across the world after its discovery in human populations in April 2009. In many countries, stringent case-based epidemiologic investigations had been introduced, allowing public health authorities to design appropriate interventions for prevention and control. However, the high transmission rates and relatively moderate course of influenza disease did not justify prolonged implementation of such measures. In the US, for example, case reporting was discontinued in July 2009, a change that was later followed in other countries. In retrospect, the accumulation of data in the initial months is contributing to a pool of useful resources for supporting epidemiologic research, which has continued to generate outputs in term of risk factor analyses, clinical guidelines development, and the establishment of public health policy.

In Hong Kong, the first imported swine flu case was reported on 1 May 2009. The first wave peaked in September 2009 with more than 4500 cases recorded per week. By the first week of November 2009, over 30,000 laboratory confirmed cases have been reported to the Department of Health of the Hong Kong Special Administrative Region Government[1]. In the first two months of May and June 2009, investigation of each laboratory confirmed case was performed actively by the Department’s Centre for Health Protection (CHP). The daily statistics, alongside residence location down to building level of each case, was uploaded on the CHP website. Active case investigation was continued through the end of September, after which mandatory testing of suspected cases stopped. In this study, we set out to explore the diffusion pattern of swine flu by examining all reported cases in the first three months, which represents therefore the very initial spread of the infection in the territory of Hong Kong, home to a 7 million population and gateway to mainland China. We reckon that an exploration of the routinely collected georeferenced data would allow epidemiologic pattern to be delineated, which would be of useful reference should another epidemic occur in the future. 

## Methods

A database was established to include all laboratory confirmed cases of swine flu reported between 1 May and 31 July 2009 in Hong Kong, Provided by the CHP, the anonymised dataset had included the age, gender and building location of each individual. Institutional approval for access to the data was sought from the Department of Health, in compliance with the Personal Data (Privacy) Ordinance.  Each textural residential address was transformed to x and y coordinates in Hong Kong Grid 1980. The geocoding led to the creation of a georeferenced dataset with point data for each swine flu patient during initial spread.

Digital maps were acquired from the Lands Department of the Hong Kong Special Administrative Region Government. Hong Kong is divided into 3 main regions – Hong Kong Island, Kowloon Peninsula and the New Territories, under which there are 18 administrative districts and 400 District Council Constituency Areas (DCCA), the latter being boundaries of subdistricts that have been created for electoral purpose, each with a population of about 17000. The geographic units at these three levels were used in the study. Statistics of the districts and DCCA at the most recent by-census in 2006 were obtained from the Census and Statistics Department. Microsoft Excel was used for data input. Statistical analysis was performed using Statistical Package for the Social Sciences version 13.0 (SPSS Inc 2004). ArcGIS 9.2 was used for mapping and exploratory spatial analysis. SaTScan™ was used for the detection and characterization of space-time cluster.

## Results

### General characteristics and distribution of swine flu case

In the first three months since the diagnosis of the first case, a total of 3675 laboratory confirmed swine flu cases were recorded, of which 3460 (94.1%) could be geocoded. The characteristics of these cases are described in table 1. Overall, the male-to-female ratio was 1.04:1. Over half of the reported cases (61.2%) were between the age of 11 and 30. As we did not have the student status of the study population, we defined “students” as those who were likely to be attending kindergartens, primary or secondary schools on a daily basis. The age of these “students” would be those between 3 and 20, which amounted to 1925 (55.60%) of the geocoded population. On a spatial scale, the distribution of the study population was heterogeneous, with a reported rate ranging from 30.70 to 91.58 per 100,000 population across the 18 districts.(table 2) Compared to the 2 other main regions, Hong Kong Island accounted for 30.43% of all reported cases, against the population of 1.55 million; compared to 26.23% ( 1.74 million population) and 43.32% (3.57 million population).(Table 1)

**Figure:**
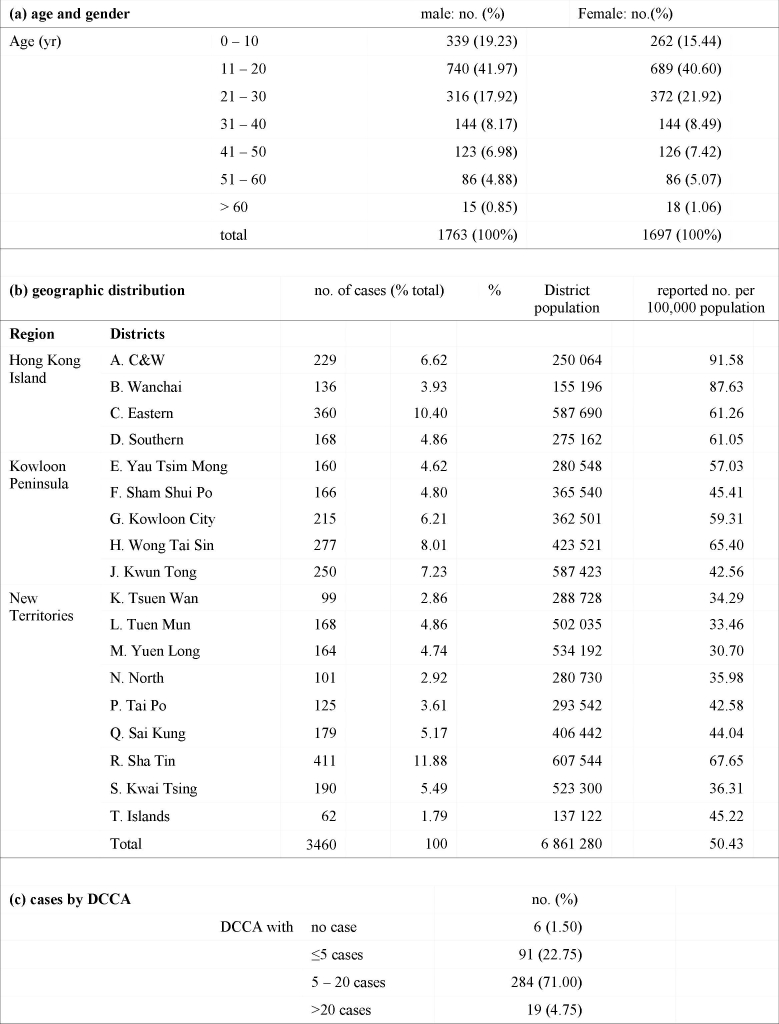
**Table 1**. Characteristics of geocoded swine flu cases reported to the Department of Health of Hong Kong, between 1 May and 31 July 2009

Almost all DCCAs (394, 98.4%) have reported at least one case over the three-month period. However, the total number of cases reported in each DCCA varied significantly. Cumulatively, only 19 DCCAs (4.75%) have reported over 20 cases. The weekly number of cases reported also varied geographically, as shown in Table 2. The increase of reports was more notable after the 5^th^ week, at around the same time that local (within Hong Kong) spread was documented. The distribution of all reported cases is shown in Figure 1, against the background of the by-census population for 2006. Apparently the number of case reports did not correlate with population density. 

**Figure:**
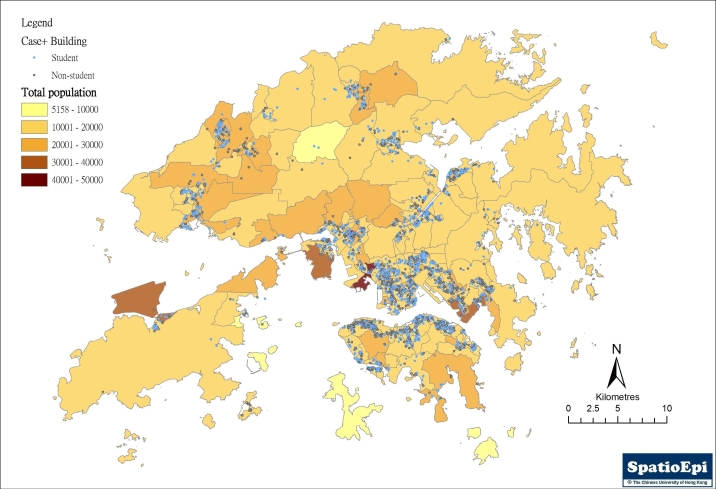
**Figure 1.** Map showing the overall distribution of reported swine flu cases (student and non-student) in the first three months of the epidemic

**Figure:**
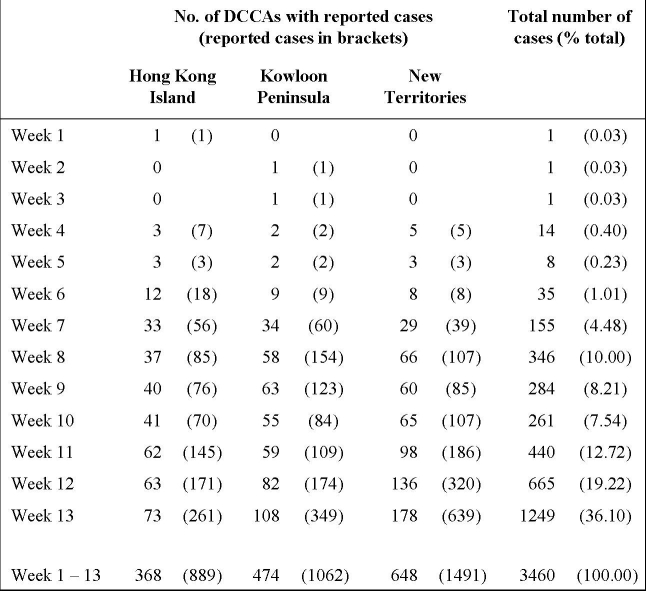
**Table 2**. Weekly reports of swine flu cases from District Council Constituency Areas (DCCA), 1 May to 31 July 2009.

### Spatio-temporal diffusion patterns

Figure 2(a) shows the diffusion of swine flu cases using an inverse distance weighting (IDW) model for interpolation. Uninhabitable areas including the harbour and mountains were excluded in the implementation of the model. The 3-month duration was divided into 9 time periods – the first covering 1 May through the end of the 5^th^ week, during which local transmission has not yet been documented, followed by 8 weekly periods. These are shown as different shade intensity on the map. While the initial cases were more localized to Hong Kong Island, separate foci could be seen in the two other regions. SaTScan™ was applied to further characterize the spatio-temporal patterns. Using the Space-time Permutation Model, and with the implementation of a 50% spatial and temporal window, 6 clusters could be identified (p<0.05). The results are shown in figure 2(b) and Table 3.  On a temporal scale, only the primary cluster on Hong Kong Island has extended from the very early phase through week five and beyond, an indication that initial local spread has occurred evidently there. The Hong Kong Island primary cluster was temporally linked to the primary clusters in Kowloon and the New Territories. The 2 Kowloon secondary clusters also followed the Hong Kong Island primary cluster in time, while the secondary cluster in the New Territories could have been one of the initial source foci, alongside the Hong Kong Island primary cluster.

**Figure:**
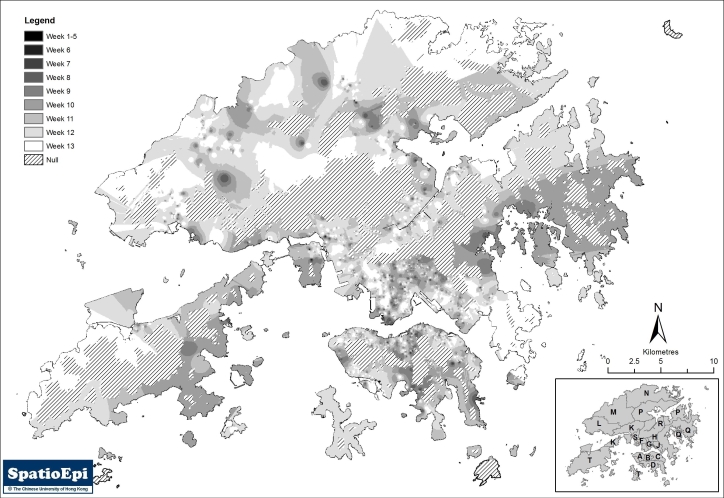
**Figure 2. **Display of the temporo-spatial patterns of swine flu spread in Hong Kong: **(a)** Diffusion of all cases by interpolation using inverse distance weighting, after exclusion of non-inhabitable areas, demonstrating situation through 9 time intervals, beginning 1 May 2009.

**Figure:**
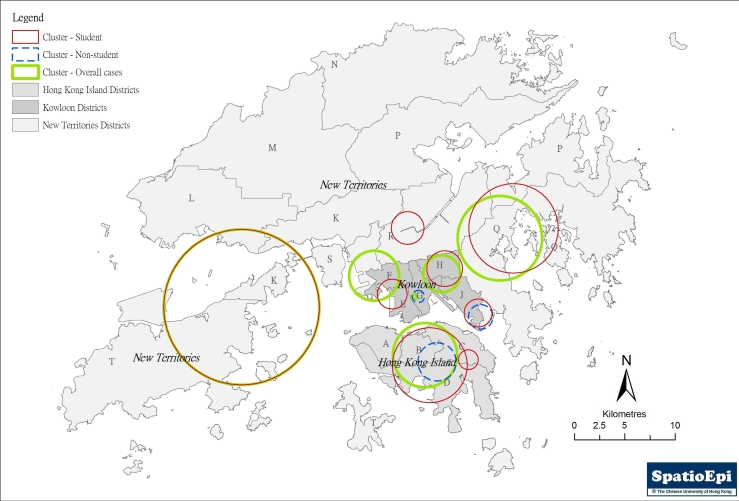
**(b)** Demonstration of temporo-spatial clusters of all cases, student cases and non-student cases using SaTScan™, with analyses made separately in the three regions of Hong Kong Island, Kowloon Peninsula and the New Territories) 

Temporo-spatial distribution was explored in the three main geographic regions after separating all cases into students and non-students.(figure 2(b)) Four otherwise unidentified clusters were revealed. Two of these were student clusters – one in the New Territories (District R) and the other on Hong Kong Island (District C), the latter partially overlapping with and temporally following the primary all-cases cluster. The other two were small non-student clusters, one overlapping spatially with a student cluster in District J in Kowloon but preceded it by one month, while the other temporally following and spatially overlapping with an all cases cluster in the same region (District G). Exploration of non-student cases did not reveal the presence of any additional unique clusters. Interpolation by IDW of non-student cases resulted in scattered foci throughout the territory, while that for students looks remarkably similar to the all-cases map.  

**Figure:**
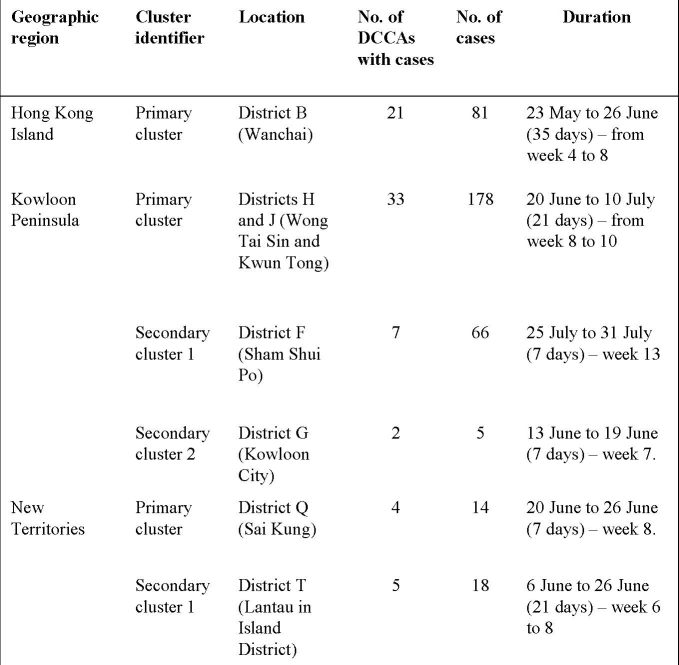
**Table 3.** Spatio-temporal clusters identified in three geographic regions in Hong Kong using SaTScan™

Using results from SaTScan™ and IDW, the paths of diffusion of swine flu cases were reconstructed, which is shown in figure 3. It can be seen that there were multiple foci of spread over this initial period of three months. The earlier foci on Hong Kong Island and Kowloon Peninsular could be related to one another, which were followed by 3 other foci in the New Territories, including Lantau Island on the west. At the end of the three-month period, the distribution of swine flu cases remained scattered.

**Figure:**
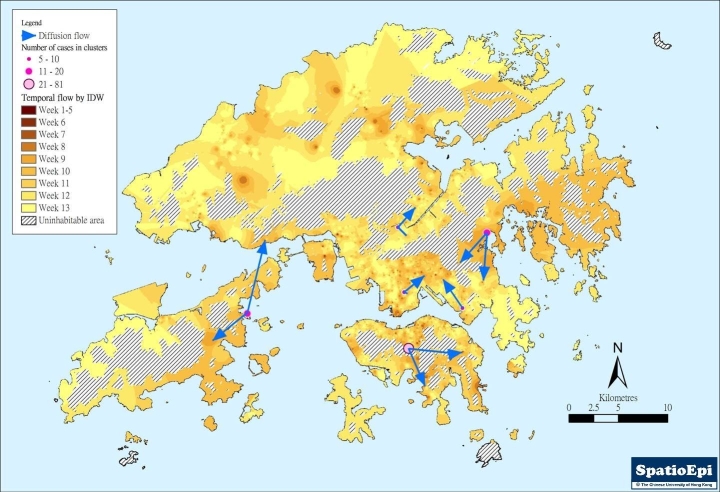
**Figure 3.** Diffusion of swine flu cases reconstructed from data obtained from temporo-spatial exploration depicted in figure 2.

## Discussion

The swine flu pandemic is now a well-characterized condition, in biologic, clinical and epidemiologic terms.[2] From the public health perspective, spatial diffusion of swine flu has, however, remained a less commonly appreciated phenomenon. Diffusion can be described as “the dynamics by which a phenomenon originally located at one point becomes transferred to another.”[3] The concept of diffusion is not restricted to public health but has also been examined in social contexts, like urbanization and social movements.[4] [5] In infectious disease epidemiology, diffusion is an important concept as it depicts the dynamics of the spread of the microorganism in time and space. Surprisingly, diffusion has only been systematically examined occasionally, in a limited number of infectious disease outbreaks, for example, measles,[3] cholera,[6] pertussis,[7] poliomyelitis,[8] typhoid[9] and H5N1 avian influenza.[10] The emergence of swine fu has provided a unique opportunity to study the diffusion of influenza virus, as has been reported on a global scale,[11] but not at national levels. Very often, epidemiology of swine flu in a country is described as a snapshot overview of the condition without further analysis on its dissemination within national boundary. In the development of national strategy, it is crucial to understand the dynamics of spread within a country so that interventions can be prioritized and rolled out strategically, at the right time and place.  It is against these backgrounds that the study was conceptualized when swine flu hit Hong Kong about a year ago.

Our study showed that swine flu has not been spreading swiftly across the territory in the initial phase since the first case was discovered. At the end of the first three month period, one fifth of the DCCAs, each with a similar population size, have reported no more than 5 cases. A combination of SaTScan™ and IDW methodologies has enabled us to highlight six initial foci with spatial diffusion on Hong Kong Island and Kowloon Peninsula, the highly urbanized regions in Hong Kong. There were smaller temporo-spatial clusters of infections beyond these densely populated areas. The relatively “slow” pace of spread supports the observations reported by other researchers that airborne transmission of swine flu was lacking.[12] The virus has presumably permeated through the population via close person-to-person contacts. The mapping of residence locations of all reported cases is therefore a valid investigative approach. Interactions within households and with neighbours in close spatial proximity should have underlain swine flu diffusion in the population. The slow diffusing pattern is in line with the relatively low basic reproductive number of 1 to 3, [13] [14] [15] compared to that of measles, an airborne virus. Interestingly, household transmission, though important, may in fact be less efficient than seasonal influenza,[16] [17] though this remains to be confirmed. By separating the geocoded cases into students and non-students, we further determined that student infections tended to be more clustered. If all student cases were excluded, the connectivity among swine flu cases in the community became very loose and might not have led to the subsequent epidemic. Students were therefore likely to be the main virus disseminators across Hong Kong, as has been reported in other countries for swine flu.[18] [19] and seasonal influenza.[20]

Our study carries certain limitations. Firstly, the data were drawn from a notification system, and therefore under-reporting was unavoidable. Specifically the milder cases could have been missed, while diagnoses of other patients might have been omitted if sampling was not performed in time. The date of diagnoses of each person had, likewise, varied considerably from one health service setting to another, an artifact which might affect the precision of any time-space analyses. However, the high volume of reported cases and the meticulous case-based investigation process introduced by the Government have served to minimize the problems arising therein.  Secondly, there was no perfect or comprehensive tool for characterizing infection diffusion. In our study, IDW turned out to be a useful model for assessing the locations of initial foci as well as the directions of diffusion, whereas SaTScan™ offered a mechanism for defining critical masses of cases in time and space. The final model, albeit a crude one, can be adapted for enabling rapid assessment of infection diffusion to be made when a virus or other microorganism becomes introduced in a population. The assessment has relied on the use of regularly collected data, their processing, followed by the application of standard GIS tools for depicting the pattern of temporo-spatial diffusion. The results can be used for prioritizing the implementation of vaccination programme, as vaccines are often in short supply in the initial phase. Our results lend support to the high heterogeneity of the pattern of swine flu diffusion, which is closely associated with population structure and mobility,[21] In practice, the diffusion pattern of an infection should be routinely delineated in a locality as an integral component of public health responses to any infectious disease threats.  

## Acknowledgements

Dr SK Chuang and Dr Thomas Tsang of Centre for Health Protection, Department of Health of the Hong Kong SAR Government, are thanked for their assistance in enabling the swine flu dataset to be available for the diffusion studies described in this report. Miss Mandy Li is thanked for her support in the creaion of the georeferenced dataset. 

## Funding information

This study is funded by the Direct Grant of the Medical Faculty, The Chinese University of Hong Kong.

## Competing interests

The authors have declared that no competing interests exist.
